# Nigeria needs a publicly accessible medical register

**DOI:** 10.1097/MS9.0000000000004600

**Published:** 2025-12-16

**Authors:** Temitomi Jane Oyedele, Nicholas Aderinto

**Affiliations:** a Department of Medicine and Surgery, Bowen University, Iwo, Nigeria; b Department of Medicine and Surgery, Ladoke Akintola University of Technology, Ogbomoso, Nigeria

*To the editor,*

Public trust in health care systems depends on transparency^[1]^. In Nigeria, however, verifying a doctor’s registration status remains unnecessarily difficult. The Medical and Dental Council of Nigeria (MDCN), established under the Medical and Dental Practitioners Act (Cap M8 LFN 2004) with the statutory mandate to regulate medical, dental, and alternative medicine practice, maintains the national register of practitioners^[2]^. However, this register is not accessible to the public. While the Council is legally responsible for determining professional standards, licensing practitioners, and safeguarding the public through credential oversight, its registration system remains outdated: the core register is still predominantly paper based, supplemented by a limited internal digital database that only MDCN staff and select government agencies can access upon formal request^[2]^. For the public, employers, hospitals, and credentialing bodies, verification requires written applications because no public-facing search portal exists. This opaque, manual-dependent system, largely unchanged since the 1980s, undermines confidence in medical regulation. The paper adheres to the TITAN guidelines^[3]^.

The consequences of this opacity have become increasingly evident. In recent years, individuals have been prosecuted for posing as medical doctors using forged or borrowed credentials^[4]^. Such cases erode public trust in medical institutions and illustrate the vulnerabilities inherent in a closed regulatory system. Even legitimate practitioners suffer, as employment, credentialing, or migration processes can be slowed by the absence of a simple, verifiable digital register. In addition, the lack of reliable, up-to-date registration data constrains national and regional health workforce planning. Accurate information about the number of registered doctors, their geographic distribution, and their specialty profiles is essential for forecasting, migration monitoring, and equitable health resource allocation^[5]^. In contrast, regulatory bodies such as the UK General Medical Council and the Rwanda Medical and Dental Council maintain publicly accessible, regularly updated online databases that permit real-time verification by patients, employers, and policymakers^[5,6]^.

Creating a secure, publicly accessible, and routinely updated digital register in Nigeria would immediately promote accountability and deter fraud while supporting ongoing national e-governance reforms. A practical pathway for achieving this vision is to adopt a phased implementation strategy. In the first phase, the MDCN could deploy a basic yet secure public search portal built on its existing internal database. Such a portal would allow verification by name or registration number, incorporate daily automated backups, and comply with data protection requirements under Nigeria’s Data Protection Act and relevant international principles. Even in its simplest form, such a portal would offer significant public benefit with relatively low technical complexity. A second phase would focus on strengthening the system against fraud. Biometric enrolment such as fingerprint or facial recognition could be integrated into registration and license renewal processes. Cryptographic technologies, including blockchain-backed verification or digital signatures, could be introduced to secure certificates and make alterations or forgeries instantly detectable. These reforms would close loopholes exploited in recent ICPC prosecutions involving credential fraud and would bring Nigeria’s regulatory infrastructure closer to international best practices.

Furthermore, more ambitious phase would transform the register into an interconnected digital platform capable of integration with hospital human-resource systems, insurance bodies such as the National Health Insurance Authority, training institutions, and regional credentialing networks. With open application programming interfaces, credential verification during hiring, facility accreditation, or specialist training could become automated and instantaneous. Such a system would also support the free movement of health workers envisioned under the African Continental Free Trade Area and broader efforts at African health workforce harmonization. The result would be a modern, fraud-resistant, continentally interoperable infrastructure rather than a static administrative ledger.

Moreover, a digitally accessible, structured, machine-readable register is the foundational substrate for next-generation AI-enabled regulatory tools. Recent advances in AI, including increasingly capable large language models with strong medical reasoning abilities, have already begun reshaping medical diagnostics, health systems administration, and public health surveillance^[7]^. In the regulatory context, AI systems could perform real-time credential verification using optical character recognition and natural language processing; detect anomalous registration patterns through graph analytics; support predictive modeling of workforce distribution and migration flows using privacy-preserving federated learning; and facilitate intelligent public interfaces allowing natural-language queries.

Public access to professional registers is not merely an administrative reform but an essential patient-safety mechanism and the gateway to modern, technology-enabled health workforce governance. Trust in medicine begins with accountability, and accountability begins with transparency.

## Ethical approval

Not applicable.

## Consent

Not applicable.

## Data Availability

No new data was generated for this study.
